# Supplementary material for: Analysis of PPARγ Signaling Activity in Psoriasis
Source: Int J Mol Sci. 2021 Aug 10;22(16):8603. doi: 10.3390/ijms22168603 (PMC8395241; doi:10.3390/ijms22168603)
Supplement: Supplementary file 1 [file ijms-22-08603-s001.zip › Supplemental materials_Analysis of PPARg signaling activity in psoriasis/Pathway models/Models images and html files/Differentiation of psoriatic T cells/111697.html]

IL27


# Protein IL27

|  |  |
| --- | --- |
| URN | urn:agi-llid:246778 |
| Total Entities | 0 |
| Connectivity | 1939 |
| Name | IL27 |
| Description | interleukin 27 |
| Notes | The protein encoded by this gene is one of the subunits of a heterodimeric cytokine complex. This protein is related to interleukin 12A (IL12A). It interacts with Epstein-Barr virus induced gene 3 (EBI3), a protein similar to interleukin 12B (IL12B), and forms a complex that has been shown to drive rapid expansion of naive but not memory CD4(+) T cells. The complex is also found to synergize strongly with interleukin 12 to trigger interferon gamma (IFNG) production of naive CD4(+) T cells. The biological effects of this cytokine are mediated by the class I cytokine receptor (WSX1/TCRR). [provided by RefSeq, Jul 2008] |
| Primary Cell Localization | Extracellular |
| Class | Ligand |

---

|  |  |
| --- | --- |
| Pathway | Th1-Cell Differentiation |
|  | AHR Signaling in Tr1 Cells Function |
|  | Eosinophil Survival by Cytokine Signaling |
|  | Dendritic Cells Function in Atherosclerosis |
|  | Th1-Cells Activation and Proliferation in Atherosclerosis |
|  | Lymphocyte Mediated Myocardial Injury in Myocarditis |
|  | Atopic Dermatitis |
|  | Proteins Involved in Psoriasis |
|  | T-Cells Differentiation Block in Psoriasis |
|  | Dendritic Cells Function in Psoriasis |
|  | T-Cell Cytotoxic Response against Melanocytes in Vitiligo |
|  | CD4+T-Cell Response in Celiac Disease |
|  | Th1-Cell Activation in Crohn's Disease |
|  | Genes with Susceptibility for Crohn's Disease and Ulcerative Colitis |
|  | Proteins Involved in Rheumatoid Arthritis |
|  | Proteins Involved in Chronic Obstructive Pulmonary Disease |
|  | Proteins Involved in Glomerulonephritis |
|  | IL27R -> STAT Signaling |
|  | C5AR1 -> Expression Targets in Lymphoid System and Blood |
|  | Elevated Receptors -> Expression Targets in Spleen |
|  | Elevated Receptors -> Expression Targets in Thymus |
|  | TLR4 -> NF-kB/IRF Expression Targets |
|  | PPAR Psoriasis |
|  | 1\_Differentiation of psoriatic T cells |
|  | 4\_1\_Polymorphisms associated with inflammatory bowel diseases |
|  | Differentiation of psoriatic T cells |

---

|  |  |
| --- | --- |
| Group | Genes with Mutations Associated with Inflammatory Bowel Diseases |

---

|  |  |
| --- | --- |
| MedScan ID | 246778 |

---

|  |  |
| --- | --- |
| LocusLink ID | 246778 |
|  | 246779 |
|  | 365368 |

---

|  |  |
| --- | --- |
| Alias | interleukin 27 |
|  | IL-27 p28 subunit |
|  | interleukin-12p35-related protein p28 |
|  | interleukin-27 receptor ligand activity |
|  | interleukin IL30 |
|  | RGD1561420 |
|  | IL30 |
|  | IL-27 subunit alpha |
|  | interleukin-27 subunit alpha |
|  | interleukin-27 receptor ligand |
|  | IL27 |
|  | interleukin IL27p28 |
|  | IL-12p35-related protein p28 |
|  | interleukin IL27 |
|  | interleukin IL-27 subunit |
|  | interleukin-27 receptor ligands |
|  | IL27p28 |
|  | IL-27A |
|  | interleukin IL-27 p28 subunit |
|  | interleukin 27A |
|  | IL-27 subunit |
|  | MGC71873 |
|  | interleukin 30 |
|  | interleukin IL-27A |
|  | OTTHUMP00000122516 |
|  | p28 |
|  | IL-27 |
|  | IL27A |
|  | IL-27-A |
|  | IL27-A |
|  | interleukin-30 |
|  | IL-27p28 |

---

|  |  |
| --- | --- |
| GO ID | 0005125 |
|  | 0045523 |
|  | 0005102 |
|  | 0006954 |
|  | 0045087 |
|  | 0070106 |
|  | 0002230 |
|  | 0045078 |
|  | 0042129 |
|  | 0045625 |
|  | 0009617 |
|  | 0005829 |
|  | 0005788 |
|  | 0005576 |
|  | 0005615 |
|  | 0002376 |
|  | 0010469 |
|  | 0050688 |

---

|  |  |
| --- | --- |
| KEGG ID | hsa:246778 |
|  | mmu:246779 |
|  | rno:365368 |

---

|  |  |
| --- | --- |
| Organism | Homo sapiens {Organism urn:agi-taxid:9606} |
|  | Mus musculus {Organism urn:agi-taxid:10090} |
|  | Rattus norvegicus {Organism urn:agi-taxid:10116} |
|  | Mus musculus |
|  | Homo sapiens |
|  | Rattus norvegicus |

---

|  |  |
| --- | --- |
| Mouse chromosome position | 7 |

---

|  |  |
| --- | --- |
| OMIM ID | 608273 |

---

|  |  |
| --- | --- |
| Rat chromosome position | 1q36 |

---

|  |  |
| --- | --- |
| Hugo ID | 19157 |
|  | HGNC:19157 |

---

|  |  |
| --- | --- |
| Human chromosome position | 16p12.1-p11.2 |
|  | 16p11 |

---

|  |  |
| --- | --- |
| Swiss-Prot Accession | Q8NEV9 |
|  | Q8NEV9.2 |
|  | Q8K3I6 |
|  | Q8K3I6.1 |
|  | A0N0L2 |
|  | Q6P676 |
|  | D3ZPW3 |

---

|  |  |
| --- | --- |
| GenBank ID | NC\_000016 |
|  | XM\_011545780 |
|  | XP\_011544082 |
|  | NM\_145659 |
|  | NP\_663634 |
|  | AC138894 |
|  | CH471279 |
|  | EAW52277 |
|  | EF064720 |
|  | ABK41903 |
|  | JA104595 |
|  | CCA63967 |
|  | AY099296 |
|  | AAM34498 |
|  | BC062422 |
|  | AAH62422 |
|  | Q8NEV9 |
|  | NC\_000073 |
|  | XM\_006507857 |
|  | XP\_006507920 |
|  | NM\_145636 |
|  | NP\_663611 |
|  | AC125169 |
|  | CH466531 |
|  | EDL17402 |
|  | AI662173 |
|  | AK152114 |
|  | BAE30958 |
|  | AK153466 |
|  | BAE32017 |
|  | AY099297 |
|  | AAM34499 |
|  | BC119402 |
|  | AAI19403 |
|  | Q8K3I6 |
|  | NC\_005100 |
|  | XM\_344962 |
|  | XP\_344963 |
|  | AC\_000069 |
|  | XM\_002725687 |
|  | XP\_002725733 |
|  | AABR07005777 |
|  | AAHX01008502 |
|  | NC\_018927 |
|  | AMYH02031790 |
|  | HQ447563 |
|  | ADQ32049 |
|  | AC\_000029 |
|  | AAHY01066400 |
|  | AC\_000148 |
|  | ABBA01043652 |
|  | AABR06008864 |
|  | NT\_010393 |
|  | NW\_001838230 |
|  | NW\_926262 |
|  | AC\_000059 |
|  | NT\_039433 |
|  | NW\_001030877 |
|  | NW\_001084773 |
|  | NW\_047562 |
|  | AABR03000083 |

---

|  |  |
| --- | --- |
| Swiss-Prot ID | IL27A\_HUMAN |
|  | IL27A\_MOUSE |
|  | D3ZPW3\_RAT |

---

|  |  |
| --- | --- |
| Cell Localization | Secreted |
|  | Extracellular |

---

|  |  |
| --- | --- |
| Ensembl ID | ENSG00000197272 |
|  | ENSP00000349365.1 |
|  | ENST00000356897.1 |
|  | ENSMUSG00000044701 |
|  | ENSMUSP00000054637.5 |
|  | ENSMUST00000058429.5 |
|  | ENSP00000349365 |
|  | ENST00000356897 |
|  | ENSMUSP00000054637 |
|  | ENSMUST00000058429 |

---

|  |  |
| --- | --- |
| MGI ID | MGI:2384409 |
|  | 2384409 |

---

|  |  |
| --- | --- |
| RGD ID | 1561420 |

---

|  |  |
| --- | --- |
| Unigene ID | Hs.528111 |
|  | Mm.222632 |

---

|  |  |
| --- | --- |
| Homologene ID | 17087 |

---

|  |  |
| --- | --- |
| Shape | Rhomb |

---

|  |  |
| --- | --- |
| IPI ID | IPI00302598 |
|  | IPI00170089 |
|  | IPI00766159 |
|  | IPI00361203 |

---
